# Genetic Correlation, Pleiotropy, and Molar Morphology in a Longitudinal Sample of Australian Twins and Families

**DOI:** 10.3390/genes13060996

**Published:** 2022-06-02

**Authors:** Kathleen S. Paul, Christopher M. Stojanowski, Toby Hughes, Alan H. Brook, Grant C. Townsend

**Affiliations:** 1 Department of Anthropology, University of Arkansas, Fayetteville, AR 72701, USA; 2 Center for Bioarchaeological Research, School of Human Evolution and Social Change, Arizona State University, Tempe, AZ 85287, USA; christopher.stojanowski@asu.edu; 3 Adelaide Dental School, The University of Adelaide, Adelaide, SA 5005, Australia; toby.hughes@adelaide.edu.au (T.H.); alan.brook@adelaide.edu.au (A.H.B.); 4 Barts and the London Dental Institute, Queen Mary University of London, London EC1M 6AX, UK

**Keywords:** quantitative genetics, molar morphology, pleiotropy, dental development

## Abstract

This study aims to expand our understanding of the genetic architecture of crown morphology in the human diphyodont dentition. Here, we present bivariate genetic correlation estimates for deciduous and permanent molar traits and evaluate the patterns of pleiotropy within (e.g., m1–m2) and between (e.g., m2–M1) dentitions. Morphology was observed and scored from dental models representing participants of an Australian twin and family study (deciduous *n* = 290, permanent *n* = 339). Data collection followed Arizona State University Dental Anthropology System standards. Genetic correlation estimates were generated using maximum likelihood variance components analysis in SOLAR v.8.1.1. Approximately 23% of deciduous variance components models and 30% of permanent variance components models yielded significant genetic correlation estimates. By comparison, over half (56%) of deciduous–permanent homologues (e.g., m^2^ hypocone–M^1^ hypocone) were significantly genetically correlated. It is generally assumed that the deciduous and permanent molars represent members of a meristic molar field emerging from the primary dental lamina. However, stronger genetic integration among m2–M1/M2 homologues than among paired deciduous traits suggests the m2 represents the anterior-most member of a “true” molar field. The results indicate genetic factors act at distinct points throughout development to generate homologous molar form, starting with the m2, which is later replaced by a permanent premolariform crown.

## 1. Introduction

When it comes to dental diversity, mammals are unrivaled. Two conditions contribute to this diversity within individuals [1,2]. Most mammals are *heterodonts*, meaning their tooth rows include multiple tooth classes, each with distinct crown form: incisors, canines, premolars, and molars [3,4]. For many mammals, dental variation is also observed across an individual’s lifespan; permanent teeth replace an initial set of deciduous teeth in a condition called *diphyodonty* [5,6,7]. The origins and underpinnings of these conditions have been considered using several lines of evidence, including embryology, experimental genetics, mathematical modeling, paleontology, and phylogenetics [6,7,8,9,10,11,12,13].

The field of quantitative genetics has also provided insight into these conditions, outlining the relative contribution of genes to tooth form and the coordination of genetic effects in determining dental phenotypes. In these efforts, pedigreed samples and relatedness coefficients approximate underlying additive genetic variation, which may (high *heritability* traits) or may not (low *heritability* traits) explain the structure of a corresponding dental data set—e.g., [14,15,16,17,18,19,20,21]. Quantitative genetic efforts also examine the potential for certain genetic effects to influence multiple dental characters (*pleiotropy*). With regard to understanding the structure of mammalian dentitions, this work has provided crucial insight into the role of genes in partitioning the tooth row into distinct modules. While many studies have focused on tooth size in human and non-human primates [22,23,24,25,26], some efforts have centered around non-metric crown variants, shape variables, and cusp configuration [17,27,28,29,30,31,32]. 

Metric patterns have suggested genetic partitioning of distinct incisor and postcanine modules that may be highly conserved across mammals [23,25,27]. Submodularity (premolar/molar) in the postcanine dentition is also consistent with predictions outlined by Butler’s morphogenetic field model [33,34,35]. However, a higher degree of genetic integration across fields has been reported for some primate populations [22,26]. The genetic architecture of human crown variation, in many cases, supports morphogenetic field effects, with the anterior-most member of each field characterized by higher heritability estimates [26] and traits expressed on neighboring teeth characterized by high genetic correlation estimates [31]. While patterns are not entirely consistent across mammalian taxa, quantitative genetic work has been essential to probing the foundations of heterodonty by examining the role of genes in structuring tooth form across the arcade.

Recent work has also focused on within-individual variation throughout ontogeny by considering the diphyodont (deciduous and permanent) dental complex as a cohesive entity. Heritability estimates for crown morphology suggest, on average, a moderately strong and stable contribution of genes to morphological variation across arcades (maxillary–mandibular) and across dentitions (deciduous–permanent), which may explain the high correspondence in crown form between primary and replacement teeth [19]. Recent findings also suggest this continuity is due, in part, to the reuse of genes throughout development to replicate crown form. 

Recently, the authors reported genetic correlations for anterior crown morphology in a longitudinal dental sample of Australian twins and families [36]. Pleiotropy was indicated for few (<10%) deciduous incisor and canine character pairs and nearly one third of permanent incisor and canine pairs. In contrast, significant genetic correlations were reported for the majority (~70%) of deciduous–permanent homologues. These results implicate genetic factors in the conservation of incisor and, especially, canine crown form across an individual’s span of dental development and the transition from deciduous to permanent teeth [36]. The findings provide important insight into the maintenance of distinct morphogenetic fields throughout crown development and tooth replacement in humans.

Yet to be explored is the genetic architecture of morphology of the diphyodont *postcanine* tooth row. Although heritability estimates have been reported for crown characters of the deciduous and permanent molars [19], genetic correlations have yet to be established. This represents a considerable gap in our understanding of dental variation, which the present study aims to address (see Research Aims and Hypotheses below). 

The postcanine tooth row is a unique region of the human diphyodont dental complex. In the anterior dentition, primary incisors and canines are shed and replaced by larger permanent elements that are nearly identical in overall shape and morphology. In contrast, the postcanine dentition consists of deciduous molars, which are shed and replaced by permanent premolars [37,38,39]. The first deciduous molars could be considered premolariform, sharing select morphological similarities with their permanent successors; the second deciduous molars, however, look like smaller versions of the first permanent molars [40,41,42]. Another important distinction between the anterior and postcanine dentitions involves their dental laminae precursors. All deciduous teeth originate from the primary dental lamina, while their permanent successors originate from the secondary dental lamina [43]. The permanent molars, however, are unique in that: (a) they are the only teeth to develop from the primary dental lamina that are not exfoliated and later replaced, and (b) they are the only permanent teeth that do not have deciduous precursors [40,44,45,46]. Therefore, it may be more appropriate to consider the deciduous and permanent molars to be part of a cohesive primary postcanine field.

### Research Aims and Hypotheses

The aim of this study is to examine the structure of additive genetic contributions to postcanine crown variation in the diphyodont dentition, with a focus on crown morphology. Here, our interest is in quantifying the degree to which the same genetic effects contribute to variation in distinct morphological characters within a sample of known relatives. To achieve this goal, we generated a series of quantitative genetic models to estimate bivariate genetic correlations (*ρ_G_*) for deciduous and permanent molar morphology. The analyses test the following null hypotheses:

1. Paired deciduous traits are genetically independent of one another:
  dtrait1–dtrait2 *ρ_G_* = 0; dtrait1–dtrait2 *ρ_G_* ≠ 1 (no pleiotropy).
2. Paired permanent traits are genetically independent of one another:
  PTrait1–PTrait2 *ρ_G_* = 0; PTrait1–PTrait2 *ρ_G_* ≠ 1 (no pleiotropy).
3. Paired deciduous/permanent traits are genetically independent of one another:
  dtrait1–PTrait1 *ρ_G_* = 0; dtrait1–PTrait1 *ρ_G_* ≠ 1 (no pleiotropy).

## 2. Materials and Methods

Materials and methods follow those outlined in Paul et al. (2021). Molar morphology data were collected for 25 deciduous crown traits and 38 permanent crown traits referencing the University of Adelaide Twin Study (UAT) dental cast collection (*n* = 290 subadults; *n* = 339 adults). The UAT represents nearly forty years of data collection with over 1200 twins and 2000 relatives (as of 2014) enrolled as participants from the regions surrounding Adelaide, South Australia and Melbourne, Victoria [47]. 

Sampling for the current study targeted individuals included in UAT Cohort 2, [47,48,49], a longitudinal subset of the broader study. Cohort 2 members were recruited through the Australia National Health and Medical Research Council’s registry of monozygotic (MZ) and dizygotic (DZ) twins; members of the twins’ immediate families were also invited to participate [47,48,49]. Dental casts for Cohort 2 represent three key phases of participants’ dental development: (1) deciduous dentition, (2) mixed dentition (i.e., a combination of deciduous and permanent teeth present in the oral cavity), and (3) permanent dentition. If the teeth of twins’ parent(s) or an older sibling were molded and casted, typically only phase 3 would be represented for those individuals. 

The present study sample includes individuals spanning over 100 nuclear families, the majority of which would be characterized as European–Australian with respect to ancestry or bioregional affiliation—see [19,36,47]. Genealogy information indicates that extended relationships (beyond nuclear family) are limited if not absent from the study. As such, kinship pairs are characterized as MZ twin, DZ twin, full-sibling, half-sibling, or parent-offspring. Individuals belonging to different families were assumed to be non-relatives, approximating randomly sampled members of a broad population—following [19,36,47].

Although UAT dental casts were assigned unique identifiers, during data collection individuals and families were recoded using a study-specific numbering system. This further preserved subject anonymity—see [19,36]. Research design and protocols were reviewed by Arizona State University’s Institutional Review Board, the University of Adelaide School of Dentistry, and the Craniofacial Biology and Dental Education Research Group (see IRB Statement).

### 2.1. Data Collection Methods

Morphological data collection followed standards outlined by the Arizona State University Dental Anthropology System (ASUDAS) [50,51]. The traits included in this study and their respective abbreviations are shown in Table 1. Per this system, each crown character’s expression was quantified as an ordinal “grade” on both left and right sides of the dentition [51]. ASUDAS reference plaques were used during data collection, and expression was scored from stone casts with the aid of supplemental lighting. While the ASUDAS was formulated for permanent morphology, these standards have been successfully employed in previous investigations of deciduous crown variation with moderately low intraobserver error rates—see Results and [19,36]. Ultimately, all M3 traits were eliminated from the study due to restricted representation of M3s in the cast sample.

### 2.2. Analytical Methods

#### 2.2.1. Observer Error

To test for intraobserver error, a subset (~20%) of the original sample was rescored eight or more weeks after initial data collection—see [19]. Raw grade differences between data recording sessions were referenced to ensure that any existing error was not systematic. Absolute grade differences were calculated [52] to examine the full magnitude of between-session error. Quantitative genetic analyses were performed on all morphological variables for which average intraobserver error fell below 0.30 grades. Results for models involving traits characterized by an error range greater than 1 grade were conservatively flagged and interpreted with caution—following [36]. 

Morphological variants were separated into three distinct variables prior to analysis: (1) left side expression, (2) right side expression, and (3) maximum expression [19,36]. For the maximum expression variable, each individual was represented by either left or right antimere data for each trait based on differential observability (whichever side is present and observable) or expression (whichever side is associated with the highest degree of trait expression).

#### 2.2.2. Quantitative Genetic Modeling

Maximum likelihood variance components analysis was used to generate a suite of parameter values, including narrow-sense heritability and bivariate genetic, environmental, and (derived) phenotypic correlations. These methods have been outlined in previous publications [18,26,31,32], with narrow-sense heritability and genetic correlations for the anterior dentition recently reported for this sample [19,36]. Variance components analysis utilizes documented genealogical and demographic data (age, sex, household) to isolate fixed and random effects while modeling the variance/covariance structure of a phenotypic data set—in this case, molar crown morphology [53,54]. For the analyses, pedigree and demographic data were obtained from UAT records. Twin pairs were assigned unique numbers with zygosity listed based on UAT documentation [47].

Bivariate genetic correlation models were generated using SOLAR v.8.1.1 [55,56], a software that models phenotypic covariance as:

Ω=G Υ 2Φ+E Υ I

where *Ω*: trait (co)variance matrix; *G*: genetic (co)variance matrix; Υ: Kronecker product operator; *Φ*: kinship matrix; *E*: environmental (co)variance matrix; and *I*: identity matrix. Parameter estimates generated for each model include genetic correlation (*ρ_G_*), environmental correlation (*ρ_E_*), and derived phenotypic correlation ($\rho_{P}$)—see [36] for $\rho_{P}$ equation. 

For each bivariate trait pair, separate models were generated for likelihood comparison: (1) all parameter values freely estimated, and (2) parameter of interest held constant (model 2a: *ρ_G_* = 0; model 2b: |*ρ_G_*| = 1). For the genetic correlation parameter, significant difference (α = 0.05) in likelihood between model 1 and model 2a is interpreted as complete pleiotropy. Significant difference in likelihood between model 1 and both model 2a and 2b is interpreted as incomplete pleiotropy—following [26,28,29,31].

For analytical purposes, ordinal variables were treated as continuous, with *inorm* data transformation applied to limit violation of distributional assumptions where appropriate—see [19,36]. This approach is justified by current understandings of ASUDAS trait expression (i.e., Liability or Threshold Models), in which morphological characters are quasi-continuous with latent genetic potential following a normal distribution and observed phenotypic variation binned into discrete categories as dictated by population-level genetic “thresholds” for expression [57,58,59,60,61,62,63,64]. Further support for this approach comes from findings that variation in tooth number and size also conforms to a quasi-continuous statistical model with thresholds. From these results a unifying model has been constructed and further developed to include tooth shape [65,66]. Several demographic covariates were considered (sex, age, and sex*age interaction) and fixed when their mean effects were deemed significant (conservatively, p ≤ 0.10) in initial variance components models of univariate heritability—see Tables S1 and S2 [19,36].

## 3. Results

### 3.1. Observer Error Results

Certain deciduous traits were removed from the study because they were monomorphic (m^1^ cusp 5) for this sample or because the traits are likely non-homologous with the permanent molar characters upon which scoring standards are based (m^1^ Carabelli’s trait, m^1^ parastyle, m_1_ anterior fovea, m_1_ protostylid, and m_1_ cusp 7) (Table 1). This is due to m1′s premolariform crown and not the specific scoring system employed. Deciduous and permanent groove pattern were also eliminated because the data structure for these traits is categorical as opposed to ordinal (Y pattern, X pattern, + pattern). 

Maximum intraobserver error ranged from 0–1 grade for the majority of traits scored. Those characterized by error outside of this range include: m^1^/M^1^ metacone, m^1^/M^1^ parastyle, M_1_ anterior fovea, m_1_/M_2_ cusp 7 (for all, maximum error = 2 grades), and m_2_ /M_2_ protostylid (maximum error = 3 grades). Of these traits, only m_2_/M_2_ protostylid were omitted from the genetic correlation analysis based on error results, as all other listed traits are associated with mean error values less than 0.30. Additionally, as reported in Paul et al. (2020), error data for m^1^ metacone (binomial probability = 0.03), M_1_ anterior fovea (binomial probability = 0.02), and M^1^ parastyle (binomial probability = 0.01) showed observation session biases—either all error session scores were lower or higher than the original scores. Results are presented for associated models but flagged for interpretive purposes (Tables S1 and S2).

### 3.2. Genetic Correlation Results

Genetic correlation results for paired antimeres are presented in Table 2. Correlation estimates are high for all traits, with many approaching or equaling 1.0. Complete pleiotropy is indicated for all left/right paired deciduous traits. Antimeric trait pairs in the permanent dentition show strong integration with few exceptions. No pleiotropy is indicated for M^2^ paracone, M_2_ cusp 6, or M_2_ distal trigonid crest. Note that models for these traits are flagged for interpretive purposes, because the standard deviation ranges for certain parameters were incalculable or problematic. As more M1s than M2s were available for data collection, these models were also characterized by relatively small sample sizes. 

For all other permanent left/right trait pairs, some degree of pleiotropy is indicated—incomplete for M^2^ Carabelli’s trait and M_1_ distal trigonid crest and complete for all other characters. These results suggest significant genetic redundancy driving bilateral symmetry in postcanine crown morphology and justify the practice of collapsing left/right paired data sets. For the subsequent intertrait genetic correlation analyses, maximum expression data were analyzed, which served to increase sample size for each model.

**Figure 1:**
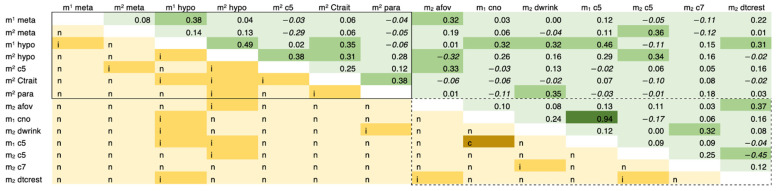
Genetic correlation matrix for deciduous molar morphology. Correlation estimates are shown above the diagonal with positive values nonitalicized and negative values italicized. Cells are shaded based on absolute estimate values (<0.3 = light green; 0.3–0.6 = green; >0.6 = dark green). Pleiotropy-based model likelihood results are shown below the diagonal (n = no pleiotropy; i = incomplete pleiotropy; c = complete pleiotropy). Cells are shaded based on degree of pleiotropy (n = light yellow; i = yellow; c = dark yellow). Model results for within-arcade trait pairs are enclosed in boxes. Maxillary trait pairs are outlined with a solid line and mandibular trait pairs are outlined with a dotted line. For trait names and abbreviations, see Table 1.

Postcanine genetic correlations were calculated within dentitions (Tables S1–S2). Deciduous results are shown in Table S1 and indicate limited genetic integration for morphology of the deciduous molars. Approximately 23% of the quantitative genetic model results are significant, which is beyond the expectations of family-wise error at α = 0.10. Most of these models show incomplete pleiotropic relationships between paired traits, with the exception of m_1_ cusp number–m_1_ cusp 5; significance testing for this model yielded values consistent with complete pleiotropy (Figure 1).

Of the trait pairs characterized by incomplete pleiotropy, nearly half are expressed on the same tooth crown: m^1^ metacone–m^1^ hypocone, m^2^ metacone–m^2^ cusp 5, m^2^ hypocone–m^2^ cusp 5, m^2^ hypocone–m^2^ Carabelli’s trait, m^2^ hypocone–m^2^ paracone, m^2^ cusp 5–m^2^ Carabelli’s trait, m^2^ Carabelli’s trait–m^2^ paracone, m_2_ anterior fovea–m_2_ distal trigonid crest, m_2_ deflecting wrinkle–m_2_ cusp 7, and m_2_ cusp 7–m_2_ distal trigonid crest. Of note, pleiotropy is indicated for one metameric pair: m^1^ hypocone–m^2^ hypocone (Figure 1).

Genetic correlations for deciduous molars are both positive and negative in value (mean *ρ_G_* = 0.11; absolute mean *ρ_G_* = 0.16) (see Table S1). While within-arcade correlation values are stable across the dentition (maxillary mean *ρ_G_* = 0.14; maxillary absolute mean *ρ_G_* = 0.19; mandibular mean *ρ_G_* = 0.12; mandibular absolute mean *ρ_G_* = 0.19), genetic correlations for morphological isomeres are weaker (maxillary/mandibular mean *ρ_G_* = 0.09; maxillary/mandibular absolute mean *ρ_G_* = 0.14). Of the significant models, genetic correlations were positive for all but three: m^2^ metacone–m^2^ cusp 5, m^2^ hypocone–m_2_ anterior fovea, and m_2_ cusp5–m_2_ distal trigonid crest (Figure 1).

**Figure 2:**
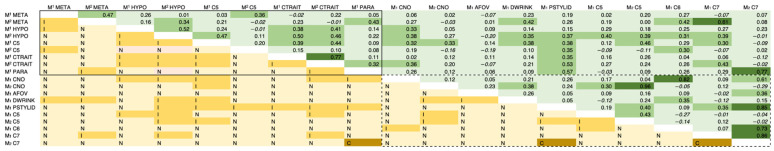
Genetic correlation matrix for permanent molar morphology. Correlation estimates are shown above the diagonal with positive values nonitalicized and negative values italicized. Cells are shaded based on absolute estimate values (<0.3 = light green; 0.3–0.6 = green; >0.6 = dark green). Pleiotropy-based model likelihood results are shown below the diagonal (N = no pleiotropy; I = incomplete pleiotropy; C = complete pleiotropy). Cells are shaded based on degree of pleiotropy (N = light yellow; I = yellow; C = dark yellow). Model results for within-arcade trait pairs are enclosed in boxes. Maxillary trait pairs are outlined with a solid line, and mandibular trait pairs are outlined with a dotted line. For trait names and abbreviations, see Table 1.

Genetic correlation results for the permanent dentition are presented in Table S2. For permanent molar morphology, 30% of models yielded significant results, which is beyond the expectations of family-wise error at α = 0.10. Three trait pairs are completely pleiotropic, and all involve cusp 7: M^1^ paracone–M_2_ cusp 7, M_1_ protostylid–M_2_ cusp 7, and M_1_ cusp 7–M_2_ cusp 7 (Figure 2). The remaining 47 significant models indicate incomplete pleiotropic relationships, many of which (19%) involve traits expressed on the same tooth crown (*M*^1^: metacone–hypocone, hypocone–cusp 5, hypocone–Carabelli’s trait, cusp 5–Carabelli’s trait; *M*^2^: hypocone–Carabelli’s trait; *M*_1_: cusp number–cusp 6, anterior fovea-deflecting wrinkle, cusp 5–cusp 6; *M*_2_: cusp number–cusp 5). 

Genetic redundancy is also indicated for the vast majority of metameric trait pairs, including: metacone, hypocone, Carabelli’s trait, cusp 5 (mandibular), and cusp 7. This is a consistent pattern, considering that genetic correlations were not estimated between neighboring teeth for paracone, cusp 6, protostylid, or distal trigonid crest, while deflecting wrinkle and anterior fovea are only scored on a single tooth. For all other significant models, the patterns appear idiosyncratic, although M^1^/M^2^ hypocone and M_1_ protostylid account for over 40% of the pleiotropic relationships (Figure 2).

Permanent molar models, like their primary counterparts, yielded both positive and negative genetic correlations (mean *ρ_G_* = 0.20; absolute mean *ρ_G_* = 0.23) (Table S2). Again, within-arcade bivariate correlation is fairly stable across the maxillary (mean *ρ_G_* = 0.22; absolute mean *ρ_G_* = 0.25) and mandibular (mean *ρ_G_* = 0.21; absolute mean *ρ_G_* = 0.26) dentitions, while these values are slightly lower for isomerically paired characters (mean *ρ_G_* = 0.19; absolute mean *ρ_G_* = 0.21). Significant models yielded positive estimates with one exception: M_1_ cusp 5–M_1_ cusp 6 (Figure 2).

The results of the deciduous–permanent genetic correlation analyses are presented in Table 3. These include only metameric m2, M1, and M2 traits, as m1 morphology more closely resembles that of the replacement tooth (permanent P1) than other postcanine elements derived from the primary dental lamina. Deciduous–permanent homologues are moderately genetically integrated, with 56% of models yielding significant results, a markedly greater percentage than in the deciduous and permanent dentitions alone. For all significant models, incomplete pleiotropy is indicated; none of the deciduous–permanent trait pairs are characterized by complete genetic redundancy. 

On average, deciduous–permanent metameres are more strongly correlated (mean *ρ_G_* = 0.36; absolute mean *ρ_G_* = 0.43). Interestingly, the results show weaker genetic correlation between maxillary deciduous–permanent homologues (mean *ρ_G_* = 0.36; absolute mean *ρ_G_* = 0.36) than between their mandibular counterparts (mean *ρ_G_* = 0.39; absolute mean *ρ_G_* = 0.50). All analyzed trait pairs are positively correlated with two exceptions: m^2^–M^2^ cusp 5 and m_2_–M_2_ cusp 5; pleiotropy is not indicated for these two models (Table 3).

## 4. Discussion

### 4.1. Morphology Versus Size

Considering both raw and absolute values, mean correlation for molar morphology (deciduous < 0.20 and permanent < 0.25) is similar to that reported for anterior dental morphology (deciduous < 0.21 and permanent < 0.23) in this sample. While this suggests stability in the degree of genetic redundancy occurring in distinct regions of the dentition, these values are markedly lower than reported averages for postcanine crown size in humans (maxillary = 0.73 and mandibular = 0.84) [26]. The same can be said for postcanine crown size correlations in callitrichid and cercopithecoid monkeys (tamarins: maxillary = 0.64 and mandibular = 0.73; macaques: maxillary = 0.50 and mandibular = 0.45; baboons: maxillary = 0.51 and mandibular = 0.46) [19,22,23,25], as well as mice (maxillary = 0.72 and mandibular = 0.72) [25]. This indicates that a greater degree of genetic redundancy underlies size data than morphology data collected from tooth crowns. This is not surprising, given the functional constraints on tooth size relating to occlusion, eruption, and the surrounding bony anatomy of the masticatory complex [67,68], although shape is not entirely exempt from these limitations [69]. It appears that genetic effects are efficiently deployed to meet these constraints—something that sets crown size apart from crown form.

Our results corroborate another reported difference between dental metric and morphological genetic architectures: genetic correlations are, with few exceptions [25], positive for metric traits but both positive and negative for morphological traits [19,22,23,26,31,32,36]. As reasoned in previous studies, this is likely due to the complex nature of crown morphology [31,32,36]. ASUDAS traits include variants that correspond with both negative and positive topographic features: cuspules, fissures, ridges, depressions, and crests, to name a few. Moreover, these features occur on various surfaces of the tooth crown: lingual, buccal, occlusal, mesial, and distal [36]. It is not surprising that the same genes are implicated in multiple phenotypic outcomes, in some cases with corresponding effects (e.g., increase expression trait 1—increase expression trait 2) and, in other cases, with opposing effects (e.g., increase expression trait 1—decrease expression trait 2). We discuss negative correlations and potential interpretations in more detail below.

### 4.2. Antimeric Correlations and Bilateral Symmetry

Genetic correlation estimates for left–right paired traits suggest strong bilateral integration for molar morphology. Most values approach 1.0, with complete pleiotropy indicated for all deciduous and most permanent traits (Table 2). With the exception of M_2_ cusp 6, all derived phenotypic correlations for left–right character pairs are positive and exceed 0.30. Together, these results affirm a tendency toward strong morphological symmetry in the molars, which—in nearly all cases—is driven by consistency in genetic effects. Similar results were obtained for anterior (incisor and canine) morphology in this sample [36] and for dental phenotypes observed in other human [26,31] and non-human primate samples [25]. These consistent findings across traits, populations and species empirically justify the standard anthropological practice of “collapsing” an individual’s left and right side dental data to a single value per trait. In fact, to skip this step in a reconstruction of evolutionary relationships would almost certainly bias the analysis because it would lead to the inclusion of genetically redundant information.

However, we note that the only other quantitative genetic study of postcanine morphology reported limited symmetry in genetic effects, as interpreted from disparate left–right heritability estimates, limited genetic correlation, and moderate derived phenotypic correlation for paired characters [32]. This may reflect a sample-specific decoupling of genetic factors across antimeres, but it is also possible the patterns reported in Stojanowski et al. (2019) are the result of dichotomizing quasi-continuous trait variation to presence/absence (i.e., calculating genetic correlations between binary traits)—an approach that was not employed in the current study.

### 4.3. Within-Dentition Correlations (Deciduous–Deciduous and Permanent–Permanent)

Considering deciduous and permanent dentitions separately, molar morphology is minimally integrated. The deciduous molar analyses identified one completely pleiotropic trait pair: m_1_ cusp number–m_1_ cusp 5. This is not surprising, given that cusp 5 variation (namely, presence versus absence) directly influences variation in cusp number [50,51,70]. Therefore, this result reflects the mechanics of scoring these interdependent traits on a crown that typically possesses either four or five cusps [71,72]. 

In the permanent dentition, all cases of complete pleiotropy involve M_2_ cusp 7. The genetic redundancy in M_1_–M_2_ cusp 7 is expected, given their metameric relationship, but the strong correlations between M_2_ cusp 7–M^1^ paracone and M_2_ cusp 7–M_1_ protostylid are difficult to interpret. These results may indicate that similar genes are involved in accessory cusp formation and crown elaboration along the buccolingual plane. Yet, if that is the case, it is unclear why similar results were not obtained for M_1_ cusp 7.

Of the remaining traits that share some degree of genetic redundancy, many occur *on the same tooth*, which suggests a high degree of within-crown morphological integration. Although this pattern reflects a true biological phenomenon—overlapping genes implicated in manifold morphological outcomes—it also underscores the limitations of our methods for quantifying crown variation. By this, we mean that ultimate crown morphology is the result of developmental processes that are highly coordinated within a single tooth: oral epithelial–ectomesenchymal interactions, determination of placode location and size, enamel knot formation and placement, inner-enamel epithelial folding, and tissue (enamel) secretion—as reviewed in [73]. 

Experimental research has shown these processes are tightly controlled by (epi)genetic mechanisms that act on tooth crowns, and indeed the entire dentition, as cohesive entities [74,75,76,77,78]. Yet, current standards for quantifying morphological variation treat individual cusp size, crest presence, tubercle formation, and fissure patterning as isolated phenomena [50,51,79]. Researchers might adjust for this statistically by reducing multivariate morphological data sets to their “essential” components using PCA or PCoA. However, the ASUDAS bins variation into discrete “grades”, for which imputation is generally not a valid approach when morphology is unobservable and data are missing [50,51]. In short, we are limited in our ability to probe the ways in which crown characters have correlated impacts on population variation because we lack methods for holistically measuring crown form. While computer automated approaches to dental phenomic profiling (e.g., geometric morphometrics, topography variables, complexity measures, sheering quotients, “fingerprinting”) might yield alternatives to the ASUDAS, to date these methods have been restricted in their anthropological application, often to functional studies of diet or wear [80,81,82,83,84,85,86,87,88].

Another set of traits that accounts for a large portion of the significant model results includes *later-forming cusps of the upper molars*, especially hypocone, cusp 5, and Carabelli’s trait. Of the deciduous traits that yielded valid model results, m^1^ hypocone shares an incomplete pleiotropic relationship with ~54% percent of them. This result for m^2^ hypocone is ~62% and ~32% for Carabelli’s trait. M^1^ hypocone shows significant genetic correlation with 39% of other permanent traits, while model results for M^2^ hypocone (56%), M^1^ cusp 5 (50%), and M^2^ Carabelli’s trait (50%) indicate incomplete pleiotropy for over half of the characters analyzed. This finding suggests that coordinated additive genetic effects play a major role in the developmental regulation of cusp size and presence in postcanine teeth.

The pattering cascade model provides a framework for interpreting these findings. It posits that cusp number and size (initially quantified as height but here quantified as area) are determined by: (a) overall size of the tooth germ, and (b) spatial patterning of enamel knots [89,90]. At the enamel–dentine junction, secondary enamel knots mark the site of future cusp tips and are surrounded by zones of inhibition, where signaling molecules regulate morphology by restricting formation of additional enamel knots [10,91,92,93,94]. In this way, homologous cusps and their positioning arise throughout crown formation via a dynamic reaction–diffusion process, where a shift in one secondary enamel knot might have a “cascade” effect altering overall tooth form [10,77,89,95]. In the maxillary molars, the cusps of the trigone (protocone, paracone, and metacone) are the earliest forming; the talon (hypocone) and accessory cusps (cusp 5, Carabelli’s trait, and paracone) form latest or, in some cases, not at all [96,97,98]. Thus, the strong correlation between hypocone and other late-forming cusps may reflect coordinated gene regulation of cusp spacing dictated by earlier forming enamel knots. This interpretation is in line with those of previous applications of the patterning cascade model to human molar morphology [99,100].

This pattern is not as strong in the lower molars, however. Interestingly, in the permanent mandibular teeth, the later forming cusps (cusp 5 and cusp 6) are significantly correlated, but the correlation is *negative*. This suggests the same genes are implicated in lower cusp 5 and cusp 6 expression but their effects are opposing. A similar result was reported by Stojanowski and colleagues in their study of human molar morphology [32]. Their interpretation invoked previous research on life history traits that suggests negative genetic correlations reflect cases of within-organism resource competition across developmental processes [101,102,103,104]. 

Combining this insight with expectations outlined in the patterning cascade model, we might interpret the negative correlation between these neighboring cusps as competition for “cellular real estate” during morphogenesis and enamel secretion [13,32,89,105]. The key word is *neighboring*. The difference between cusp 5 and cusp 6 in the lower molars and most later-forming cusps of the upper molars is that cusps 5 and 6 are situated next to each other at the distal-most aspect of the crown. Thus, their sizes depend directly on relative enamel organ partitioning and the cellular territory allotted to their neighbor during crown formation [32]. The nature of this relationship is somewhat intuitive and reflected in data collection standards for cusp 6—this is one of the few traits for which expression is based on size *relative* to another feature, in this case cusp 5 [50,51]. What these genetic correlation results provide, however, is greater insight into how development and (epi)genetic factors work in tandem to produce elaborate, multicuspid crown forms from fairly simple epithelial precursors.

### 4.4. Between-Dentition Correlations (Deciduous–Permanent)

As the UAT Cohort 2 sample is longitudinal with most individuals represented by deciduous and permanent dental casts, this study provides a unique opportunity to examine the morphological architecture of the diphyodont molar row. Because m1s are more similar in crown form to permanent premolars, we generated genetic correlations for homologous trait pairs of m2–M1 and m2–M2. 

Raw and absolute genetic correlations are higher for paired deciduous–permanent molar traits (mean = 0.36 and absolute mean = 0.43) than for paired traits within dentitions (deciduous: mean = 0.11 and absolute mean = 0.16; permanent: mean = 0.20 and absolute mean = 0.23), with over half characterized by incomplete pleiotropy. This is a similar pattern to that observed in the anterior dentition for this sample, and it is tempting to draw a similar interpretation: that there is “a stronger genetic mechanism for morphological conservation across the diphyodont dentition than within individual tooth rows” [36]. However, we must consider the unique developmental trajectory shared by deciduous and permanent molars. 

Unlike the incisors and canines, the permanent molar class is not succedaneous. That is to say, deciduous molars are replaced not by permanent molars but by permanent premolars. Permanent molars do not replace any primary elements and are the only permanent teeth to arise from the primary dental lamina—the same dental lamina from which all deciduous teeth originate [98]. Unlike incisors and canines, deciduous molars and their premolar successors have limited morphological variation in common, at least with respect to variants quantified using ASUDAS standards [50,51]. For this reason, it is more appropriate to treat deciduous and permanent molars as metameres: homologous structures of a single postcanine tooth row or molar field [106]—following [33,40,41,44,107].

That said, the stronger genetic correlation between m2 and the permanent molars corroborates previous research that suggests the m1 is, for lack of a better term, an oddity. The m1 is *somewhat* premolariform, but, compared to other elements, it does not strongly resemble any other tooth in the diphyodont dental complex. If considered part of a meristic molar field, this unique morphology is unexpected [40,44,96,108,109]. Further, despite its early crown completion, m1 is more variable in size and morphology than is m2 [110,111,112]. Previous researchers have suggested that m2 represents the “key tooth” of a cohesive molar field or, at a minimum, the deciduous postcanine field [40,111,112,113]. While these results do not provide direct support for this statement, the relatively weak genetic correlations between m1 and m2 traits indicate the molar field might contain only four “true” members: m2, M1, M2, and M3 [41,42,44]. However, we note that M3 data were ultimately omitted from the study due to sample size limitations.

### 4.5. Study Limitations

While we note several consistencies between these and previous quantitative genetic findings, sample composition and certain methodological choices may have impacted ultimate results. First, the reported genetic correlations represent a primarily European–Australian sample characterized by its own population-specific morphology profile. Previous research has shown morphological trends in both additive and non-additive genetic factors (for example, dominance for traits contributing to crown “complexity” and mass) that may result in disparate patterns for populations of distinct biogeographic origin [114]. 

Due to the UAT’s recruitment strategy, this sample also includes a disproportionately large number of MZ twins, which sets it apart from broader pedigree/family study samples. Finally, as in our study of anterior morphology [36], morphological correlations were calculated from maximum expression (left or right) data for ordinal variables. It is possible that genetic correlations calculated from dichotomized presence/absence data for the same characters might provide additional insight into the underlying genetic architecture of these quasi-continuous traits.

## 5. Conclusions

Within dentition (deciduous–deciduous and permanent–permanent) correlation values are, on average, lower than those estimated for homologous traits across dentitions (e.g., m^2^ hypocone–M^1^ hypocone). Further, a greater percentage of deciduous–permanent trait pairs exhibit some degree of redundancy in genetic effects (incomplete pleiotropy). As both the deciduous and permanent molars arise from the primary dental lamina, they likely represent elements of a meristic tooth district. Still, the relative degree of genetic integration noted for m2–M1–M2 morphology as compared to m1–m2 morphology aligns with previous suggestions that the m2 is the anterior-most member of a cohesive primary molar field. This is compatible with the overall distinctiveness of the m1 crown form.

## Figures and Tables

**Table 1:** Morphological crown traits for UAT data collection.

Morphological Trait ^1^	Abbreviation	Dental Elements Scored
Metacone	META	m^1^, m^2^, M^1^, M^2^, M^3^
Hypocone	HYPO	m^1^, m^2^, M^1^, M^2^, M^3^
Cusp 5 (*Metaconule*) ^2^	C5	m^1^, m^2^, M^1^, M^2^, M^3^
Carabelli’s Trait ^2^	CTRAIT	m^1^, m^2^, M^1^, M^2^, M^3^
Parastyle ^2^	PARA	m^1^, m^2^, M^1^, M^2^, M^3^
Anterior Fovea ^2^	AFOV	m_1_, m_2_, M_1_
Deflecting Wrinkle	DWRINK	m_2_, M_1_
Cusp 5 (*Hypoconulid*)	C5	m_1_, m_2_, M_1_, M_2_, M_3_
Cusp 6	C6	m_1_, m_2_, M_1_, M_2_, M_3_
Cusp 7 ^2^	C7	m_1_, m_2_, M_1_, M_2_, M_3_
Cusp Number	CNO	m_1_, m_2_, M_1_, M_2_, M_3_
Groove Pattern	GROOVE	m_2_, M_1_, M_2_, M_3_
Protostylid ^2^	PSTYLID	m_1_, m_2_, M_1_, M_2_, M_3_
Distal Trigonid Crest	DTCREST	m_2_, M_1_, M_2_, M_3_

^1^ Maxillary and mandibular arcades are indicated by superscripts and subscripts, respectively. ^2^ Scoring standards were augmented for observation on the first deciduous molar, but data were ultimately removed due to lack of precision in data recording. These m1 traits are not necessarily homologous to the permanent characters upon which the scoring standards are based.

**Table 2:** Antimeric variance component correlations.

		Genetic			Environmental		Phenotypic
Trait ^1^	*N*/Cov ^2^	*ρ_G_* ^3^	*P*(*ρ_G_* = 0) ^4^	*P*(|*ρ_G_*| = 1) ^4^	*ρ_E_* ^5^	*P* (*ρ_E_* = 0) ^4^	*ρ_P_* ^6^
DECIDUOUS (l−r)							
m^1^ meta ^e^	249	0.978 ± 0.077 **	**<0.001**	0.387	−0.144 ± 0.131	0.283	0.597
m^2^ meta	278/a	0.852 ± 0.094 **	**<0.001**	0.057	−0.144 ± 0.111	0.208	0.434
m^1^ hypo ^w^	251	1.000 ± − **	**<0.001**	−	0.114 ± 0.106	0.282	0.642
m^2^ hypo	279	0.992 ± 0.056 **	**<0.001**	0.445	0.005 ± 0.112	0.967	0.648
m^2^ c5	272/s	0.868 ± 0.087 **	**<0.001**	0.070	0.132 ± 0.140	0.345	0.625
m^2^ ctrait ^w^	276	1.000 ± − **	**<0.001**	−	0.227 ± 0.112	**0.046**	0.758
m^2^ para	278	1.000 ± − **	**<0.001**	−	0.263 ± 0.117	**0.021**	0.638
m_2_ afov	268	0.753 ± 0.139 **	**<0.001**	0.053	0.182 ± 0.120	0.134	0.446
m_2_ dwrink	261	1.000 ± − **	**<0.001**	−	−0.152 ± 0.118	0.228	0.607
m_1_ c5	239/s	1.000 ± − **	**<0.001**	−	−0.085 ± 0.125	0.501	0.578
m_2_ c5	281/a, a*s	0.875 ± 0.176 **	**0.001**	0.249	0.173 ± 0.120	0.161	0.412
m_1_ c6 ^w^	−	−	**−**	−	^−^	−	−
m_2_ c6 ^cf^	−	−	**−**	−	^−^	−	−
m_2_ c7	283	0.938 ± 0.054 **	**<0.001**	0.120	0.107 ± 0.121	0.374	0.687
m_1_ cno ^w^	239/s	1.000 ± − **	**<0.001**	−	0.020 ± 0.126	0.871	0.591
m_2_ cno ^cf^	−	−	**^−^**	−	^−^	−	−
m_2_ dtcrest ^w^	253/a	1.000 ± − **	**<0.001**	−	0.269 ± 0.185	0.179	0.492
**PERMANENT (L−R)**							
M^1^ META ^E^	327	1.000 ± − **	**<0.001**	−	0.221 ± 0.095	**0.028**	0.555
M^2^ META	151/S, A*S	0.990 ± 0.232 **	**0.002**	0.483	0.544 ± 0.127	**<0.001**	0.668
M^1^ HYPO	319/S	1.000 ± − **	**<0.001**	−	−0.206 ± 0.108	0.073	0.620
M^2^ HYPO	112	1.000 ± − **	**<0.001**	−	−0.523 ± 0.312	0.276	0.878
M^1^ C5	292/A, A*S	0.968 ± 0.051 **	**<0.001**	0.263	−0.085 ± 0.143	0.558	0.675
M^2^ C5 ^W^	117	1.000 ± − **	**<0.001**	−	−0.498 ± 0.220	0.095	0.596
M^1^ CTRAIT	302/A, A*S	0.965 ± 0.032 **	**<0.001**	0.119	0.438 ± 0.100	**<0.001**	0.801
M^2^ CTRAIT	135/A, A*S	0.792 ± 0.100 *	**<0.001**	**0.023**	0.056 ± 0.257	0.828	0.641
M^1^ PARA ^E^	314	0.886 ± 0.110 **	**<0.001**	0.148	−0.429 ± 0.118	**0.001**	0.306
M^2^ PARA ^W^	154	0.093 ± −	1.000	0.500	0.440 ± −	**<0.001**	0.439
M_1_ AFOV ^E^	294/A, A*S	1.000 ± − **	**<0.001**	−	−0.098 ± 0.155	0.528	0.655
M_1_ DWRINK	301	0.973 ± 0.059 **	**<0.001**	0.324	−0.278 ± 0.112	**0.023**	0.580
M_1_ PSTYLID	293/S	0.916 ± 0.100 **	**<0.001**	0.207	0.282 ± 0.158	0.103	0.605
M_1_ C5	280/ALL	0.935 ± 0.046 *	**<0.001**	0.062	−0.149 ± 0.131	0.275	0.696
M_2_ C5 ^W^	145/S	1.000 ± − **	**<0.001**	−	0.290 ± 0.249	0.283	0.784
M_1_ C6	281/A*S	1.000 ± − **	**<0.001**	−	0.152 ± 0.114	0.183	0.512
M_2_ C6 ^W^	144	−0.075 ± −	1.000	0.500	−0.018 ± −	0.878	−0.018
M_1_ C7	330/S	1.000 ± − **	**<0.001**	−	0.144 ± 0.134	0.263	0.694
M_2_ C7 ^E^	187	1.000 ± − **	**0.047**	−	0.182 ± 0.152	0.269	0.368
M_1_ CNO	293	1.000 ± − **	**<0.001**	−	0.233 ± 0.111	**0.039**	0.588
M_2_ CNO	140	0.991 ± 0.092 **	**<0.001**	0.462	−0.144 ± 0.308	0.653	0.695
M_1_ DTCREST ^W^	300/S	0.327 ± − *	**<0.001**	**<0.001**	1.000 ± −	**<0.001**	0.338
M_2_ DTCREST ^W^	182	0.900 ± −	1.000	1.000	0.920 ± −	1.000	0.920

^1^ l/L = left; r/R = right; m/M = molar. Maxillary and mandibular traits indicated by superscript and subscript, respectively. Deciduous and permanent indicated by lowercase and uppercase script, respectively. For a list of morphological trait abbreviations, see Table 1. “e/E” superscript indicates a trait that was originally flagged for intra-observer error because the error range exceeded a single grade but whose mean error does not exceed 0.300. Traits with mean error exceeding 0.300 were omitted from the correlation analyses. All third molar traits were omitted from the correlation analyses due to sample size limitations. “w/W” superscript indicates models that are suspect due to standard deviation ranges for certain estimates. “cf/CF” superscript indicates model convergence failure. ^2^ Covariates fixed in the genetic correlation models based on univariate model results (see Paul et al., 2020). “a/A” = age; “s/S” = sex; “a*s/A*S” = age/sex interaction; all/ALL = all covariates. ^3^ Maximum-likelihood estimate of genetic correlation. Cases of incomplete pleiotropy indicated by a single asterisk. Cases of complete pleiotropy indicated by two asterisks. Dashes are associated with incalculable parameter estimates. ^4^ Probability of hypothesis (as indicated in parentheses) given pedigree structure with values *p* < 0.050 bolded. Dashes are associated with incalculable parameter estimates. ^5^ Maximum-likelihood estimate of environmental correlation. Dashes are associated with incalculable parameter estimates. ^6^ Maximum-likelihood estimate of derived phenotypic correlation. Dashes are associated with incalculable parameter estimates.

**Table 3:** Variance components correlations: paired deciduous and permanent morphology.

		Genetic			Environmental		Phenotypic
Trait ^1^	*N*/Cov ^2^	*ρ_G_* ^3^	*P* (*ρ_G_* = 0) ^4^	*P* (|*ρ_G_*| = 1) ^4^	*ρ_E_* ^5^	*P* (*ρ_E_* = 0) ^4^	*ρ_P_* ^6^
Meta (m^2^−M^1^) ^E^	355/Y	0.189 ± 0.158	0.230	**<0.001**	0.099 ± 0.148	0.502	0.147
Meta (m^2^−M^2^)	344/Y	0.183 ± 0.186	0.315	**<0.001**	−0.239 ± 0.155	0.142	0.003
Hypo (m^2^−M^1^)	352/N	0.597 ± 0.089 *	**<0.001**	**<0.001**	0.126 ± 0.140	0.370	0.460
Hypo (m^2^−M^2^)	332/N	0.445 ± 0.143 *	**0.006**	**<0.001**	−0.128 ± 0.441	0.776	0.346
C5 (m^2^−M^1^)	349/Y	0.547 ± 0.132 *	**<0.001**	**<0.001**	−0.323 ± 0.133	**0.032**	0.234
C5 (m^2^−M^2^)	326/Y	−0.157 ± 0.175	0.372	**<0.001**	0.483 ± 0.181	**0.034**	−0.062
CTrait (m^2^−M^1^)	354/Y	0.635 ± 0.075 *	**<0.001**	**<0.001**	0.040 ± 0.131	0.760	0.491
Ctrait (m^2^−M^2^)	337/N	0.368 ± 0.147 *	**0.019**	**<0.001**	−0.340 ± 0.186	0.099	0.253
Para (m^2^−M^1^) ^E^	356/Y	0.239 ± 0.122	0.057	**<0.001**	0.265 ± 0.124	**0.043**	0.243
AFov (m_2_−M_1_)^E^	329/Y	0.691 ± 0.139 *	**<0.001**	**0.032**	−0.077 ± 0.138	0.583	0.353
DWrink (m_2_−M_1_)	332/N	0.520 ± 0.078 *	**<0.001**	**<0.001**	−0.016 ± 0.158	0.920	0.440
Pstylid (m_2_−M_1_) ^E^	340/Y	0.659 ± 0.128 *	**<0.001**	**0.009**	−0.300 ± 0.132	**0.038**	0.300
C5 (m_2_−M_1_)	343/Y	0.168 ± 0.165	0.329	**0.008**	0.195 ± 0.138	0.170	0.146
C5 (m_2_−M_2_)	335/Y	−0.391 ± 0.240	0.109	**0.018**	0.502 ± 0.202	**0.050**	0.042
C7 (m_2_−M_1_)	352/Y	0.649 ± 0.154 *	**<0.001**	**0.027**	−0.234 ± 0.136	0.107	0.265
C7 (m_2_−M_2_) ^E^	345/Y	0.455 ± 0.240	0.056	0.071	−0.096 ± 0.214	0.657	0.180

^1^ m/M = molar. Maxillary and mandibular traits indicated by superscript and subscript, respectively. Deciduous and permanent traits indicated by lowercase and uppercase script, respectively. All traits represented by their maximum antimeric expression score. For a list of morphological trait abbreviations, see Table 1. “E” superscript indicates a trait that was originally flagged for intra-observer error because the error range exceeded a single grade but whose mean error does not exceed 0.300. Traits with mean error exceeding 0.300 were omitted from the correlation analyses. All models involving m_2_/M_2_ cusp 6, m_2_ cusp number, M^2^ paracone, and M_1_/M_2_ distal trigonid crest either failed to converge or yielded suspect results due to standard deviation ranges for parameter estimates and are excluded from the table and all summary statistics. ^2^ Covariates fixed in the genetic correlation models based on univariate model results (see Paul et al., 2020). Only sex was fixed for deciduous–permanent homologue correlations due to the structure of the “age” data set. “N” = sex covariate not fixed; “Y” = sex covariate fixed. ^3^ Maximum-likelihood estimate of genetic correlation. Cases of incomplete pleiotropy indicated by a single asterisk. Dashes are associated with incalculable parameter estimates. ^4^ Probability of hypothesis (as indicated in parentheses) given pedigree structure with values *p* < 0.050 bolded. Dashes are associated with incalculable parameter estimates. ^5^ Maximum-likelihood estimate of environmental correlation. Dashes are associated with incalculable parameter estimates. ^6^ Maximum-likelihood estimate of derived phenotypic correlation. Dashes are associated with incalculable parameter estimates.

## Data Availability

Phenotypic data are included as supplementary materials (details in Appendix A). Pedigree and demographic data are available upon request only due to IRB/ethics restrictions. Access to pedigree and demographic data require prior human research ethics approval or exemption by the Office of Research Ethics, Compliance and Integrity at the University of Adelaide, as well as the permission of University of Adelaide Twin Sample curators due to the sensitive nature of the data.

## Supplementary Materials

The following are available online at https://www.mdpi.com/article/10.3390/genes13060996/s1, Table S1: Variance components inter-trait correlations: deciduous morphology, Table S2: Variance components inter-trait correlations: permanent morphology. Morphology data for variables included in the analysis can be found in the attached .csv files “Deciduous Morphology Data” and “Permanent Morphology Data”.

## Appendix A

Data were collected following ASUDAS standards (see Supplementary Materials Data .csv files) [51]. Scoring standards for certain traits include “half” grades (e.g., 3.5 for metacone and hypocone and 1A for cusp 7). These “half” grades were treated as full grades for the analysis. For example, metacone expression scores traditionally range from 0–5 [51]. In this data set, scores range from 0–6 because 3.5 is treated as a full grade. Data are presented for left (l/L) and right (r/R) sides and maximum antimeric expression (max/MAX). Maxillary traits are indicated with “x/X”, and mandibular traits are indicated with “n/N”.
