# Supplementary material for: Genetic Correlation, Pleiotropy, and Molar Morphology in a Longitudinal Sample of Australian Twins and Families
Source: Genes (Basel). 2022 Jun 2;13(6):996. doi: 10.3390/genes13060996 (PMC9222655; doi:10.3390/genes13060996)
Supplement: Supplementary file 1 [file genes-13-00996-s001.zip › Table S1.pdf]

Table S1. Variance components inter-trait correlations: deciduous morphology.

| Trait [a] | N/Cov[b] | Genetic $\rho_G$[c] | Genetic $P(\rho_G=0)$[d] | Genetic $P(\lvert\rho_G\rvert=1)$[d] | Environmental $\rho_E$[e] | Environmental $P(\rho_E=0)$[d] | Phenotypic $\rho_P$[f] |
|---|---|---|---|---|---|---|---|
| $m^1$ meta[e] | | | | | | | |
| $m^2$ meta | 280/s | 0.080±0.122 | 0.515 | **<0.001** | -0.072±0.139 | 0.605 | 0.031 |
| $m^1$ hypo | 252 | 0.384±0.133* | **0.005** | **<0.001** | -0.113±0.123 | 0.262 | 0.186 |
| $m^2$ hypo | 281 | 0.041±0.116 | 0.726 | **<0.001** | 0.027±0.142 | 0.851 | 0.037 |
| $m^2$ c5 | 278/s | -0.030±0.141 | 0.832 | **<0.001** | -0.027±0.138 | 0.842 | -0.029 |
| $m^2$ ctrait | 279 | 0.056±0.106 | 0.594 | **<0.001** | -0.204±0.136 | 0.148 | <0.001 |
| $m^2$ para | 281 | -0.036±0.125 | 0.777 | **<0.001** | -0.150±0.132 | 0.267 | -0.074 |
| $m_2$ afov | 276 | 0.315±0.161 | 0.060 | **0.001** | -0.092±0.134 | 0.496 | 0.129 |
| $m_1$ cno | 261/s | 0.033±0.124 | 0.788 | **<0.001** | -0.068±0.133 | 0.609 | 0.001 |
| $m_2$ dwrink | 274 | -0.001±0.118 | 0.996 | **<0.001** | 0.051±0.138 | 0.714 | 0.013 |
| $m_1$ c5 | 260/s | 0.116±0.117 | 0.326 | **<0.001** | -0.029±0.131 | 0.822 | 0.071 |
| $m_2$ c5 | 284/a, a*s | -0.047±0.201 | 0.814 | **0.009** | 0.081±0.133 | 0.545 | 0.019 |
| $m_2$ c7 | 284 | -0.106±0.111 | 0.339 | **<0.001** | 0.021±0.134 | 0.874 | -0.072 |
| $m_2$ dtcrest | 276 | 0.224±0.113 | 0.055 | **<0.001** | 0.009±0.139 | 0.949 | 0.156 |
| $m^2$ meta | | | | | | | |
| $m^1$ hypo | 280/s | 0.136±0.140 | 0.324 | **<0.001** | -0.007±0.126 | 0.579 | 0.053 |
| $m^2$ hypo | 279/s | 0.134±0.108 | 0.218 | **<0.001** | -0.059±0.121 | 0.628 | 0.075 |
| $m^2$ c5 | 279/s | -0.287±0.139* | **0.045** | **<0.001** | -0.050±0.133 | 0.708 | -0.190 |
| $m^2$ ctrait | 279/s | 0.064±0.101 | 0.527 | **<0.001** | -0.241±0.126 | 0.069 | -0.004 |
| $m^2$ para | 281/s | -0.046±0.118 | 0.699 | **<0.001** | 0.087±0.121 | 0.475 | 0.001 |
| $m_2$ afov | 285/a, s | 0.193±0.175 | 0.268 | **<0.001** | -0.096±0.137 | 0.486 | 0.055 |
| $m_1$ cno | 281/s | 0.061±0.129 | 0.641 | **<0.001** | 0.195±0.134 | 0.156 | 0.106 |
| $m_2$ dwrink | 284/s | -0.041±0.121 | 0.732 | **<0.001** | 0.156±0.143 | 0.285 | 0.014 |
| $m_1$ c5 | 281/a, s | 0.114±0.121 | 0.350 | **<0.001** | 0.060±0.132 | 0.649 | 0.096 |
| $m_2$ c5 | 286/all | 0.359±0.203 | 0.066 | **0.021** | -0.052±0.125 | 0.676 | 0.127 |
| $m_2$ c7 | 286/s | -0.124±0.106 | 0.245 | **<0.001** | 0.059±0.124 | 0.636 | -0.073 |
| $m_2$ dtcrest | 285/s | 0.010±0.123 | 0.935 | **<0.001** | -0.062±0.141 | 0.662 | -0.014 |

| | | | | | | | |
|---|---|---|---|---|---|---|---|
| $m^1$ hypo | | | | | | | |
| $m^2$ hypo | 281 | 0.490±0.123* | **<0.001** | **<0.001** | -0.075±0.126 | 0.556 | 0.272 |
| $m^2$ c5 | 279/s | 0.019±0.159 | 0.904 | **<0.001** | 0.161±0.127 | 0.213 | 0.084 |
| $m^2$ ctrait | 280 | 0.346±0.111* | **0.004** | **<0.042** | 0.127±0.134 | 0.347 | 0.267 |
| $m^2$ para | 281 | -0.063±0.144 | 0.663 | **<0.001** | 0.160±0.125 | 0.210 | 0.030 |
| $m_2$ afov | 276/a | 0.011±0.191 | 0.956 | **<0.001** | -0.064±0.129 | 0.619 | -0.029 |
| $m_1$ cno | 262/s | 0.321±0.149* | **0.031** | **<0.001** | -0.140±0.124 | 0.268 | 0.135 |
| $m_2$ dwrink | 275 | 0.324±0.126* | **0.013** | **<0.001** | -0.086±0.134 | 0.522 | 0.181 |
| $m_1$ c5 | 261/s | 0.459±0.127* | **0.001** | **<0.001** | -0.135±0.121 | 0.272 | 0.231 |
| $m_2$ c5 | 284/a, a*s | -0.108±0.219 | 0.621 | **0.008** | 0.028±0.124 | 0.823 | -0.026 |
| $m_2$ c7 | 284 | 0.147±0.123 | 0.240 | **<0.001** | 0.031±0.127 | 0.807 | 0.105 |
| $m_2$ dtcrest | 277 | 0.309±0.134* | **0.020** | **<0.001** | -0.186±0.130 | 0.165 | 0.109 |
| $m^2$ hypo | | | | | | | |
| $m^2$ c5 | 279/s | 0.380±0.116* | **0.002** | **<0.001** | -0.249±0.121 | 0.053 | 0.165 |
| $m^2$ ctrait | 280 | 0.306±0.092* | **0.001** | **<0.065** | -0.108±0.129 | 0.407 | 0.219 |
| $m^2$ para | 281 | 0.278±0.108* | **0.012** | **<0.001** | -0.097±0.120 | 0.425 | 0.155 |
| $m_2$ afov | 286/a | -0.323±0.157* | **0.033** | **0.001** | 0.328±0.121 | **0.014** | -0.044 |
| $m_1$ cno | 282/s | 0.258±0.137 | 0.055 | **<0.001** | -0.220±0.139 | 0.129 | 0.104 |
| $m_2$ dwrink | 285 | 0.158±0.108 | 0.145 | **<0.001** | -0.029±0.141 | 0.837 | 0.111 |
| $m_1$ c5 | 282/s | 0.288±0.117* | **0.017** | **<0.001** | -0.001±0.139 | 0.992 | 0.200 |
| $m_2$ c5 | 287/a, a*s | 0.344±0.174* | **0.050** | **0.009** | -0.062±0.130 | 0.639 | 0.132 |
| $m_2$ c7 | 287 | 0.159±0.100 | 0.115 | **<0.001** | -0.120±0.124 | 0.341 | 0.090 |
| $m_2$ dtcrest | 286 | -0.023±0.112 | 0.838 | **<0.001** | -0.027±0.132 | 0.839 | -0.024 |
| $m^2$ c5 | | | | | | | |
| $m^2$ ctrait | 279/s | 0.251±0.133* | **0.050** | **<0.001** | -0.111±0.150 | 0.449 | 0.137 |
| $m^2$ para | 281/s | 0.118±0.148 | 0.422 | **<0.001** | 0.080±0.126 | 0.524 | 0.102 |
| $m_2$ afov | 285/a, s | 0.326±0.207 | 0.116 | **0.003** | -0.146±0.139 | 0.304 | 0.075 |
| $m_1$ cno | 277/s | -0.033±0.149 | 0.827 | **<0.001** | 0.095±0.142 | 0.508 | 0.017 |
| $m_2$ dwrink | 283/s | 0.129±0.142 | 0.368 | **<0.001** | 0.241±0.163 | 0.154 | 0.160 |
| $m_1$ c5 | 277/s | -0.021±0.138 | 0.878 | **<0.001** | 0.097±0.134 | 0.475 | 0.024 |
| $m_2$ c5 | 287/all | 0.056±0.232 | 0.808 | **0.006** | 0.003±0.130 | 0.980 | 0.024 |

| | | | | | | | |
|---|---|---|---|---|---|---|---|
| $m_2$ c7 | 287/s | 0.054±0.128 | 0.678 | **<0.001** | 0.068±0.135 | 0.616 | 0.057 |
| $m_2$ dtcrest | 285/s | 0.162±0.139 | 0.249 | **<0.001** | 0.020±0.166 | 0.903 | 0.106 |
| $m^2$ ctrait | | | | | | | |
| $m^2$ para | 281 | 0.378±0.107* | **0.001** | **<0.001** | -0.202±0.122 | 0.111 | 0.221 |
| $m_2$ afov | 285/a | -0.060±0.144 | 0.675 | **<0.001** | -0.001±0.145 | 0.997 | -0.035 |
| $m_1$ cno | 279/s | -0.060±0.114 | 0.599 | **<0.001** | 0.166±0.141 | 0.248 | -0.008 |
| $m_2$ dwrink | 284 | -0.016±0.100 | 0.870 | **<0.001** | -0.074±0.149 | 0.620 | -0.027 |
| $m_1$ c5 | 279/s | 0.072±0.105 | 0.497 | **<0.001** | 0.101±0.134 | 0.454 | 0.077 |
| $m_2$ c5 | 286/a, a*s | -0.099±0.170 | 0.556 | **0.007** | 0.057±0.150 | 0.706 | -0.031 |
| $m_2$ c7 | 286 | 0.075±0.093 | 0.421 | **<0.001** | 0.196±0.132 | 0.153 | 0.096 |
| $m_2$ dtcrest | 285 | -0.020±0.104 | 0.849 | **<0.001** | 0.232±0.153 | 0.149 | 0.034 |
| $m^2$ para | | | | | | | |
| $m_2$ afov | 286/a | 0.005±0.171 | 0.976 | **<0.001** | 0.127±0.123 | 0.313 | 0.064 |
| $m_1$ cno | 282/s | -0.109±0.132 | 0.415 | **<0.001** | 0.126±0.133 | 0.351 | -0.028 |
| $m_2$ dwrink | 286 | 0.348±0.112* | **0.003** | **<0.001** | -0.203±0.124 | 0.115 | 0.183 |
| $m_1$ c5 | 283/s | -0.025±0.127 | 0.847 | **<0.001** | 0.050±0.130 | 0.699 | 0.001 |
| $m_2$ c5 | 288/a, a*s | -0.012±0.198 | 0.952 | **0.005** | 0.064±0.126 | 0.612 | 0.028 |
| $m_2$ c7 | 287 | 0.181±0.109 | 0.099 | **<0.001** | 0.013±0.122 | 0.912 | 0.129 |
| $m_2$ dtcrest | 287 | 0.031±0.116 | 0.791 | **<0.001** | -0.105±0.124 | 0.402 | -0.016 |
| $m_2$ afov | | | | | | | |
| $m_1$ cno | 276/a, s | 0.099±0.183 | 0.591 | **<0.001** | -0.075±0.156 | 0.633 | 0.018 |
| $m_2$ dwrink | 274/a | 0.082±0.173 | 0.649 | **<0.001** | 0.373±0.121 | **0.005** | 0.189 |
| $m_1$ c5 | 275/a, s | 0.127±0.169 | 0.455 | **<0.001** | -0.048±0.146 | 0.743 | 0.045 |
| $m_2$ c5 | 284/a, a*s. | 0.110±0.326 | 0.727 | **0.029** | -0.138±0.122 | 0.276 | -0.050 |
| $m_2$ c7 | 284/a | 0.030±0.150 | 0.840 | **<0.001** | 0.109±0.129 | 0.403 | 0.057 |
| $m_2$ dtcrest | 277/a | 0.366±0.161* | **0.019** | **0.003** | -0.140±0.128 | 0.282 | 0.123 |
| $m_1$ cno | | | | | | | |
| $m_2$ dwrink | 272/s | 0.244±0.133 | 0.059 | **<0.001** | -0.368±0.132 | **0.014** | 0.067 |
| $m_1$ c5 | 240/s | 0.940±0.041** | **<0.001** | 0.078 | 0.624±0.081 | **<0.001** | 0.826 |
| $m_2$ c5 | 283/all | -0.167±0.213 | 0.451 | **0.005** | 0.007±0.160 | 0.967 | -0.073 |
| $m_2$ c7 | 284/s | 0.058±0.113 | 0.610 | **<0.001** | 0.157±0.130 | 0.240 | 0.084 |

| | | | | | | | |
|---|---|---|---|---|---|---|---|
| $m_2$ dtcrest | 271/s | 0.155±0.119 | 0.206 | **<0.003** | 0.244±0.138 | 0.091 | 0.184 |
| $m_2$ dwrink | | | | | | | |
| $m_1$ c5 | 271/s | 0.124±0.124 | 0.308 | **<0.001** | -0.140±0.143 | 0.335 | 0.051 |
| $m_2$ c5 | 283/a, a*s | -0.001±0.199 | 0.994 | **0.006** | -0.039±0.144 | 0.787 | -0.016 |
| $m_2$ c7 | 284 | 0.321±0.099* | **0.002** | **<0.001** | -0.011±0.139 | 0.935 | 0.246 |
| $m_2$ dtcrest | 268 | 0.082±0.115 | 0.472 | **<0.001** | -0.181±0.131 | 0.178 | 0.010 |
| $m_1$ c5 | | | | | | | |
| $m_2$ c5 | 283/all | 0.090±0.202 | 0.655 | **0.006** | 0.001±0.148 | 0.993 | 0.041 |
| $m_2$ c7 | 284/s | 0.088±0.108 | 0.414 | **<0.001** | 0.106±0.126 | 0.401 | 0.093 |
| $m_2$ dtcrest | 271/s | -0.041±0.118 | 0.731 | **<0.001** | 0.221±0.133 | 0.111 | 0.042 |
| $m_2$ c5 | | | | | | | |
| $m_2$ c7 | 285/a, a*s | 0.246±0.169 | 0.141 | **0.006** | -0.261±0.127 | 0.055 | 0.019 |
| $m_2$ dtcrest | 283/a, a*s | -0.453±0.174* | **0.009** | **0.016** | 0.150±0.124 | 0.238 | -0.138 |
| $m_2$ c7 | | | | | | | |
| $m_2$ dtcrest | 284 | 0.120±0.104 | 0.253 | **<0.001** | 0.009±0.126 | 0.941 | 0.090 |

[a]m=molar. Maxillary and mandibular traits indicated by superscript and subscript, respectively. All traits represented by their maximum antimeric expression score. For a list of morphological trait abbreviations, see Table 1. "e" superscript indicates a trait that was originally flagged for intra-observer error because the error range exceeded a single grade, but whose mean error does not exceed 0.300. Traits with mean error exceeding 0.300 were omitted from the correlation analyses. All models involving $m_1$/$m_2$ cusp 6 and all but one model involving $m_2$ cusp number either failed to converge or yielded suspect results due to standard deviation ranges for parameter estimates and are excluded from the table. These traits are omitted from Figure 1 and all summary statistics. Results for these models can be found in the supplemental files of Paul (2017).

[b]Covariates fixed in the genetic correlation models based on univariate model results (see Paul et al., 2020). "a"=age; "s"=sex; "a*s"=age/sex interaction; "all"=all covariates.

[c]Maximum-likelihood estimate of genetic correlation. Cases of incomplete pleiotropy indicated by a single asterisk. Cases of complete pleiotropy indicated by two asterisks. Dashes are associated with incalculable parameter estimates.

[d]Probability of hypothesis (as indicated in parentheses) given pedigree structure with values p<0.050 bolded. Dashes are associated with incalculable parameter estimates.

[e]Maximum-likelihood estimate of environmental correlation. Dashes are associated with incalculable parameter estimates.

[f]Maximum-likelihood estimate of derived phenotypic correlation. Dashes are associated with incalculable parameter estimates.
