# Supplementary material for: Genetic Correlation, Pleiotropy, and Molar Morphology in a Longitudinal Sample of Australian Twins and Families
Source: Genes (Basel). 2022 Jun 2;13(6):996. doi: 10.3390/genes13060996 (PMC9222655; doi:10.3390/genes13060996)
Supplement: Supplementary file 1 [file genes-13-00996-s001.zip › Table S2.pdf]

Table S2. Variance components inter-trait correlations: permanent morphology.

Trait [a]	N/Cov [b]	Genetic			Environmental		Phenotypic
		ρ_G [c]	$P(\rho_G=0)$ [d]	$P(\|\rho_G\|=1)$ [d]	ρ_E [e]	$P(\rho_E=0)$ [d]	ρ_P [f]
M^1 META[E]							
M^2 META	330	0.470±0.178*	**0.030**	**<0.001**	0.385±0.133	**0.013**	0.424
M^1 HYPO	308/A	0.264±0.125*	**0.050**	**<0.001**	-0.059±0.133	0.657	0.136
M^2 HYPO	330	0.009±0.210	0.964	**<0.001**	0.548±0.173	**0.022**	0.135
M^1 C5	308/A, A*S	0.027±0.159	0.864	**<0.001**	0.123±0.143	0.395	0.062
M^2 C5	329	0.361±0.171*	**0.033**	**<0.001**	-0.075±0.139	**0.013**	0.140
M^1 CTRAIT	308/A, A*S	-0.020±0.145	0.891	**<0.001**	0.093±0.119	0.441	0.030
M^2 CTRAIT	330	0.215±0.207	0.300	**<0.001**	-0.194±0.229	0.414	0.071
M^1 PARA	330	0.052±0.139	0.708	**<0.001**	-0.079±0.122	0.518	-0.002
M_1 CNO	330	0.063±0.184	0.733	**<0.001**	-0.070±0.124	0.574	-0.007
M_2 CNO	328	0.019±0.218	0.931	**<0.001**	-0.215±0.205	0.302	-0.075
M_1 AFOV	314/A, A*S	-0.071±0.139	0.611	**<0.001**	0.143±0.138	0.310	0.007
M_1 DWRINK	335	0.234±0.117*	**0.050**	**<0.001**	-0.005±0.178	0.979	0.150
M_1 PSTYLID	330/S	0.187±0.181	0.312	**<0.001**	0.004±0.127	0.974	0.093
M_1 C5	310/A*S	0.020±0.133	0.883	**<0.001**	0.330±0.121	**0.012**	0.111
M_2 C5	328	0.195±0.195	0.335	**<0.001**	-0.182±0.208	0.381	0.044
M_1 C6	330	0.272±0.190	0.154	**<0.001**	-0.141±0.121	0.251	0.047
M_1 C7	335/S	-0.067±0.205	0.745	**<0.001**	-0.013±0.127	0.918	-0.037
M_2 C7	329	0.067±0.355	0.848	**0.045**	-0.043±0.165	0.796	-0.003
M^2 META							
M^1 HYPO	303/A	0.160±0.194	0.420	**0.001**	0.309±0.191	0.136	0.213
M^2 HYPO	171	0.339±0.161	0.061	**<0.001**	0.130±0.277	0.643	0.252
M^1 C5	294/A, A*S	0.210±0.188	0.282	**<0.001**	-0.111±0.186	0.557	0.090
M^2 C5	171	-0.019±0.155	0.903	**<0.001**	0.369±0.250	0.195	0.031
M^1 CTRAIT	306/A, A*S	0.230±0.188	0.223	**<0.001**	-0.200±0.172	0.257	0.049
M^2 CTRAIT	177	-0.012±0.199	0.952	**<0.001**	0.384±0.222	0.124	0.100
M^1 PARA[E]	316	0.427±0.184*	**0.022**	**0.005**	-0.125±0.193	0.521	0.198

M_1 CNO	303	0.269±0.238	0.245	**0.005**	-0.070±0.142	0.625	0.088
M_2 CNO	183	-0.026±0.201	0.896	**<0.001**	-0.020±0.194	0.920	-0.023
M_1 AFOV[E]	304/A, A*S	0.010±0.212	0.964	**<0.001**	0.298±0.231	0.229	0.108
M_1 DWRINK	324	0.422±0.164*	**0.010**	**0.030**	-0.541±0.171	**0.038**	0.151
M_1 PSTYLID	312/S	0.261±0.200	0.206	**<0.001**	-0.268±0.159	0.102	0.009
M_1 C5	284/A*S	0.186±0.136	0.187	**<0.001**	-0.060±0.160	0.709	0.107
M_2 C5	183	-0.001±0.186	0.997	**<0.001**	0.029±0.185	0.876	0.011
M_1 C6	304	0.420±0.276	0.112	**0.028**	-0.082±0.142	0.564	0.136
M_1 C7	333/S	0.614±0.221*	**0.011**	**0.042**	-0.305±0.178	0.100	0.130
M_2 C7[E]	197	0.081±0.358	0.820	**0.049**	0.090±0.184	0.629	0.085
M^1 HYPO							
M^2 HYPO	302/A	0.515±0.139*	**0.001**	**<0.001**	-0.101±0.296	0.735	0.404
M^1 C5	304/A, A*S	0.237±0.116*	**0.037**	**<0.001**	-0.118±0.138	0.399	0.136
M^2 C5	302/A	-0.009±0.151	0.951	**<0.001**	-0.153±0.386	0.699	-0.021
M^1 CTRAIT	307/A, A*S	0.379±0.093*	**<0.001**	**<0.001**	0.029±0.122	0.813	0.273
M^2 CTRAIT	303/A	0.414±0.129*	**0.004**	**<0.001**	0.110±0.229	0.633	0.345
M^1 PARA[E]	308/A	0.137±0.103	0.186	**<0.001**	-0.220±0.120	0.078	0.038
M_1 CNO	305/A	0.327±0.141*	**0.022**	**<0.001**	-0.056±0.133	0.678	0.164
M_2 CNO	301/A	0.052±0.152	0.732	**<0.001**	0.261±0.200	0.218	0.107
M_1 AFOV[E]	311/A, A*S	0.086±0.104	0.416	**<0.001**	0.134±0.142	0.348	0.098
M_1 DWRINK	312/A	0.141±0.096	0.144	**<0.001**	-0.021±0.238	0.930	0.111
M_1 PSTYLID	308/A, S	0.150±0.135	0.261	**<0.001**	0.061±0.139	0.659	0.116
M_1 C5	305/A, A*S	0.292±0.087*	**0.002**	**<0.001**	-0.054±0.134	0.692	0.218
M_2 C5	301/A	0.175±0.142	0.224	**<0.001**	0.182±0.199	0.373	0.177
M_1 C6	306/A	0.254±0.147	0.080	**<0.001**	-0.052±0.133	0.697	0.122
M_1 C7	312/A, S	0.273±0.156	0.080	**0.001**	-0.121±0.132	0.369	0.096
M_2 C7[E]	301/A	0.232±0.276	0.413	**0.048**	-0.075±0.225	0.741	0.073
H^2 HYPO							
M^1 C5	293/A, A*S	0.474±0.116*	**0.001**	**<0.001**	0.214±0.188	0.270	0.425
M^2 C5	131	0.106±0.112	0.346	**<0.001**	-0.053±0.332	0.873	0.098
M^1 CTRAIT	304/A, A*S	0.504±0.134*	**0.003**	**<0.001**	0.145±0.422	0.736	0.418

M^2 CTRAIT	161	0.463±0.112*	**<0.001**	**<0.001**	-0.254±0.262	0.362	0.377
M^1 PARA[E]	315	0.218±0.117	0.079	**<0.001**	0.108±0.225	0.635	0.195
M_1 CNO	303	0.375±0.158*	**0.030**	**<0.001**	-0.415±0.274	0.209	0.186
M_2 CNO	160	0.266±0.158	0.077	**0.001**	-0.228±0.249	0.379	0.170
M_1 AFOV[E]	302/A, A*S	-0.198±0.186	0.291	**<0.001**	0.580±0.312	0.222	-0.080
M_1 DWRINK	321	0.348±0.111*	**0.003**	**<0.001**	-0.552±0.194	**0.045**	0.277
M_1 PSTYLID	310/S	0.367±0.146*	**0.023**	**<0.001**	0.040±0.216	0.853	0.265
M_1 C5	284/A*S	0.397±0.123*	**0.005**	**<0.001**	0.111±0.197	0.575	0.363
M_2 C5	162	0.389±0.131*	**0.003**	**<0.001**	-0.211±0.220	0.375	0.271
M_1 C6	304	0.314±0.164	0.070	**<0.001**	-0.453±0.207	0.079	0.129
M_1 C7	333/S	0.338±0.168*	**0.028**	**0.001**	-0.470±0.215	0.086	0.159
M_2 C7[E]	190	-0.006±0.236	0.979	**0.037**	-0.043±0.308	0.888	-0.013
M^1 C5							
M^2 C5	293/A, A*S	0.196±0.137	0.158	**<0.001**	-0.025±0.240	0.917	0.168
M^1 CTRAIT	307/A, A*S	0.390±0.108*	**0.001**	**<0.001**	-0.009±0.135	0.947	0.268
M^2 CTRAIT	293/A, A*S	0.438±0.126*	**0.002**	**<0.001**	-0.086±0.193	0.660	0.325
M^1 PARA[E]	306/A, A*S	0.088±0.109	0.429	**<0.001**	0.231±0.131	0.089	0.127
M_1 CNO	304/A, A*S	0.324±0.145*	**0.037**	**<0.001**	0.004±0.142	0.978	0.193
M_2 CNO	293/A, A*S	0.334±0.158*	**0.036**	**<0.001**	-0.090±0.180	0.618	0.219
M_1 AFOV[E]	312/A, A*S	0.143±0.119	0.223	**<0.001**	-0.310±0.157	0.071	0.031
M_1 DWRINK	314/A, A*S	0.384±0.105*	**<0.001**	**<0.001**	-0.562±0.162	**0.023**	0.217
M_1 PSTYLID	306/ALL	0.377±0.132*	**0.009**	**<0.001**	0.104±0.144	0.470	0.274
M_1 C5	303/A, A*S	0.121±0.096	0.215	**<0.001**	0.216±0.139	0.133	0.139
M_2 C5	293/A, A*S	0.456±0.142*	**0.002**	**<0.001**	-0.143±0.169	0.406	0.292
M_1 C6	303/A, A*S	0.285±0.151	0.068	**<0.001**	0.024±0.140	0.865	0.175
M_1 C7	314/ALL	0.300±0.167	0.112	**<0.001**	0.076±0.174	0.664	0.199
M_2 C7[E]	294/ALL	-0.085±0.269	0.753	**0.046**	0.166±0.205	0.421	0.029
M^2 C5							
M^1 CTRAIT	304/A, A*S	0.153±0.158	0.328	**<0.001**	-0.243±0.428	0.590	0.096
M^2 CTRAIT	163	0.100±0.111	0.374	**<0.001**	0.433±0.239	0.132	0.119
M^1 PARA[E]	314	0.081±0.117	0.488	**<0.001**	-0.059±0.235	0.803	0.063

M_1 CNO	302	0.189±0.176	0.276	**<0.001**	-0.224±0.430	0.621	0.103
M_2 CNO	162	-0.158±0.122	0.202	**<0.001**	0.599±0.171	**0.018**	-0.086
M_1 AFOV^E	302/A, A*S	-0.185±0.160	0.259	**<0.001**	0.020±0.437	0.964	0.159
M_1 DWRINK	321	0.099±0.110	0.374	**<0.001**	-0.030±0.304	0.922	0.092
M_1 PSTYLID	310/S	0.352±0.160*	**0.026**	**<0.001**	-0.915±0.040	**<0.001**	0.156
M_1 C5	283/A*S	-0.086±0.120	0.478	**<0.001**	0.084±0.362	0.818	-0.073
M_2 C5	165	-0.110±0.121	0.365	**<0.001**	0.949±0.026	**<0.001**	-0.030
M_1 C6	303	0.302±0.170	0.077	**<0.001**	-0.141±0.301	0.646	0.181
M_1 C7	333/S	-0.071±0.184	0.698	**<0.001**	0.014±0.296	0.963	-0.043
M_2 C7^E	189	0.017±0.233	0.942	**0.040**	-0.092±0.445	0.838	-0.004
M^1 CTRAIT							
M^2 CTRAIT	306/A, A*S	0.769±0.106*	**<0.001**	**0.011**	-0.308±0.177	0.115	0.482
M^1 PARA^E	309/A, A*S	0.110±0.110	0.323	**<0.001**	0.047±0.122	0.702	0.089
M_1 CNO	309/A, A*S	0.019±0.151	0.903	**<0.001**	0.159±0.129	0.224	0.079
M_2 CNO	305/A, A*S	0.123±0.155	0.430	**<0.001**	0.279±0.182	0.149	0.168
M_1 AFOV^E	314/A, A*S	0.114±0.109	0.307	**<0.001**	0.039±0.141	0.779	0.092
M_1 DWRINK	315/A, A*S	0.143±0.110	0.198	**<0.001**	-0.144±0.292	0.639	0.086
M_1 PSTYLID	309/ALL	0.350±0.134*	**0.010**	**<0.001**	-0.113±0.121	0.356	0.160
M_1 C5	308/A, A*S	0.160±0.097	0.105	**<0.001**	0.035±0.132	0.790	0.127
M_2 C5	305/A, A*S	0.259±0.148	0.089	**<0.001**	0.084±0.184	0.648	0.205
M_1 C6	309/A, A*S	0.037±0.149	0.806	**<0.001**	0.185±0.123	0.140	0.100
M_1 C7	314/ALL	0.055±0.162	0.737	**<0.001**	0.126±0.125	0.319	0.086
M_2 C7^E	304/A, A*S	-0.118±0.274	0.665	**0.050**	0.087±0.185	0.639	-0.007
M^2 CTRAIT							
M^1 PARA^E	316	0.324±0.127*	**0.013**	**<0.001**	-0.321±0.192	0.135	0.182
M_1 CNO	305	0.364±0.152*	**0.021**	**<0.001**	-0.137±0.159	0.398	0.197
M_2 CNO	173	0.204±0.145	0.188	**<0.001**	0.252±0.185	0.192	0.212
M_1 AFOV^E	303/A, A*S	-0.068±0.189	0.715	**<0.001**	0.492±0.224	0.098	0.061
M_1 DWRINK	323	0.213±0.122	0.083	**<0.001**	-0.333±0.287	0.317	0.147
M_1 PSTYLID	310/S	0.534±0.148*	**0.001**	**0.001**	-0.360±0.174	0.070	0.270
M_1 C5	285/A*S	0.268±0.111*	**0.020**	**<0.001**	0.016±0.182	0.930	0.225

M_2 C5	174	0.235±0.132	0.096	**<0.001**	0.277±0.175	0.133	0.242
M_1 C6	306	0.258±0.171	0.146	**<0.001**	-0.043±0.161	0.789	0.148
M_1 C7	335/S	0.432±0.195*	**0.037**	**0.002**	-0.312±0.209	0.181	0.166
M_2 C7^E	194	-0.015±0.280	0.958	**0.038**	0.028±0.274	0.920	0.002
M^1 PARA^E							
M_1 CNO	326	0.263±0.131*	**0.046**	**<0.001**	-0.050±0.124	0.687	0.137
M_2 CNO	315	0.115±0.155	0.456	**<0.001**	-0.131±0.190	0.500	0.043
M^1 AFOV^E	313/A, A*S	0.063±0.104	0.546	**<0.001**	-0.128±0.132	0.338	0.015
M^1 DWRINK	337	0.094±0.089	0.290	**<0.001**	-0.048±0.148	0.746	0.069
M_1 PSTYLID	325/S	0.565±0.146*	**<0.001**	**0.004**	-0.317±0.138	**0.039**	0.215
M_1 C5	305/A*S	-0.028±0.094	0.764	**<0.001**	0.148±0.129	0.260	0.009
M_2 C5	315	0.087±0.140	0.534	**<0.001**	-0.097±0.182	0.599	0.034
M_1 C6	326	0.259±0.133	0.055	**<0.001**	-0.023±0.123	0.852	0.140
M_1 C7	336/S	0.286±0.152	0.061	**<0.001**	0.001±0.131	0.993	0.153
M_2 C7	316	0.769±0.248**	**0.002**	0.210	-0.270±0.177	0.160	0.227
M_1 CNO							
M_2 CNO	300	0.124±0.188	0.511	**<0.001**	-0.047±0.159	0.770	0.055
M_1 AFOV^E	309/A*S	0.052±0.146	0.720	**<0.001**	-0.184±0.132	0.175	-0.033
M^1 DWRINK	324	0.211±0.154	0.217	**<0.001**	-0.468±0.211	0.221	0.031
M_1 PSTYLID	320/S	0.261±0.195	0.173	**0.001**	-0.061±0.133	0.651	0.097
M_1 C5	284/A*S	0.166±0.127	0.194	**<0.001**	0.048±0.143	0.736	0.117
M_2 C5	300	0.037±0.179	0.835	**<0.001**	-0.015±0.156	0.924	0.016
M_1 C6	296	0.818±0.058*	**<0.001**	**<0.001**	0.857±0.034	**<0.001**	0.838
M_1 C7	330/S	0.091±0.219	0.676	**<0.001**	-0.072±0.141	0.612	0.002
M_2 C7^E	304	0.607±0.434	0.088	0.248	-0.043±0.133	0.745	0.161
M_2 CNO							
M_1 AFOV^E	300/A, A*S	0.234±0.154	0.148	**<0.001**	0.258±0.253	0.336	0.240
M_1 DWRINK	318	0.383±0.136*	**0.007**	**<0.001**	-0.065±0.375	0.864	0.295
M_1 PSTYLID	306/S	0.241±0.182	0.205	**<0.001**	-0.206±0.191	0.295	0.079
M_1 C5	283/A*S	0.298±0.129*	**0.025**	**<0.001**	-0.048±0.174	0.782	0.224
M_2 C5	145	0.963±0.021*	<**0.001**	**0.028**	0.797±0.061	**<0.001**	0.911

M_1 C6	300	-0.052±0.195	0.791	**<0.001**	-0.011±0.159	0.943	-0.034
M_1 C7	322/S	0.115±0.217	0.597	**<0.001**	-0.180±0.199	0.373	-0.012
M_2 C7^E	187	-0.292±0.273	0.279	0.055	0.241±0.211	0.270	-0.024
M_1 AFOV^E							
M_1 DWRINK	302/A*S	0.258±0.079*	**0.002**	**<0.001**	0.042±0.141	0.766	0.224
M_1 PSTYLID	312/ALL	0.053±0.136	0.698	**<0.001**	0.135±0.150	0.374	0.078
M_1 C5	308/A, A*S	0.092±0.095	0.338	**<0.001**	0.033±0.135	0.807	0.081
M_2 C5	301/A, A*S	0.163±0.152	0.292	**<0.001**	0.220±0.240	0.381	0.177
M_1 C6	309/A, A*S	0.091±0.146	0.529	**<0.001**	-0.222±0.128	0.096	-0.023
M_1 C7	312/ALL	-0.019±0.154	0.900	**<0.001**	-0.013±0.147	0.929	-0.016
M_2 C7	303/A, A*S	0.358±0.341	0.317	0.088	-0.300±0.286	0.375	0.042
M_1 DWRINK							
M_1 PSTYLID	325/S	0.049±0.129	0.707	**<0.001**	0.246±0.196	0.247	0.083
M_1 C5	308/A*S	-0.116±0.088	0.187	**<0.001**	0.010±0.256	0.967	-0.101
M_2 C5	320	0.236±0.125	0.070	**<0.001**	0.308±0.254	0.285	0.236
M_1 C6	325	0.354±0.169	0.062	**0.004**	-0.469±0.199	0.164	0.099
M_1 C7	331/S	-0.115±0.129	0.356	**<0.001**	0.680±0.086	**<0.001**	0.086
M_2 C7^E	325	0.150±0.279	0.617	0.055	-0.191±0.456	0.716	0.032
M_1 PSTYLID							
M_1 C5	302/S, A*S	0.190±0.130	0.136	**<0.001**	-0.252±0.138	0.085	0.057
M_2 C5	307/S	0.396±0.151*	**0.016**	**<0.001**	-0.327±0.184	0.100	0.150
M_1 C6	320/S	0.088±0.195	0.650	**<0.001**	0.076±0.134	0.572	0.082
M_1 C7	331/S	0.350±0.228	0.121	**0.005**	-0.067±0.134	0.619	0.114
M_2 C7^E	313/S	0.849±0.262**	**0.003**	0.290	-0.097±0.140	0.491	0.270
M_1 C5							
M_2 C5	283/A*S	0.434±0.114*	**0.001**	**<0.001**	-0.033±0.169	0.843	0.335
M_1 C6	283/A*S	-0.272±0.123*	**0.035**	**<0.001**	-0.107±0.141	0.449	-0.200
M_1 C7	311/S, A*S	-0.006±0.151	0.966	**<0.001**	0.020±0.184	0.912	0.002
M_2 C7^E	283/A*S	-0.040±0.214	0.852	0.055	0.040±0.184	0.828	-0.006
M_2 C5							
M_1 C6	300	-0.135±0.187	0.470	**<0.001**	0.028±0.156	0.855	-0.064

M_1 C7	332/S	0.121±0.196	0.537	**<0.001**	-0.097±0.182	0.594	0.024
M_2 $C7^E$	188	-0.016±0.247	0.948	**0.039**	-0.027±0.205	0.895	-0.020
M_1 C6							
M_1 C7	330/S	0.067±0.227	0.767	**<0.001**	-0.005±0.140	0.969	0.026
M_2 $C7^E$	304	0.730±0.502	0.064	0.324	-0.085±0.129	0.516	0.152
M_1 C7							
M_2 $C7^E$	332/S	0.856±0.345**	**0.020**	0.344	-0.206±0.163	0.211	0.152

[a]M=molar. Maxillary and mandibular traits indicated by superscript and subscript, respectively. All traits represented by their maximum antimeric expression score. For a list of morphological trait abbreviations, see Table 1. “E” superscript indicates a trait that was originally flagged for intra-observer error because the error range exceeded a single grade, but whose mean error does not exceed 0.300. Traits with mean error exceeding 0.300 were omitted from the correlation analyses. All third molar traits were omitted from the correlation analyses due to sample size limitations. All models involving M^2 paracone, M_2 cusp 6, and M_1/M_2 distal trigonid crest either failed to converge or yielded suspect results due to standard deviation ranges for parameter estimates and are excluded from the table. These traits are omitted from Figure 2 and all summary statistics. Results for these models can be found in the supplemental files of Paul (2017).

[b]Covariates fixed in the genetic correlation models based on univariate model results (see Paul et al., 2020). “A”=age; “S”=sex; “A*S”=age/sex interaction; “ALL”=all covariates.

[c]Maximum-likelihood estimate of genetic correlation. Cases of incomplete pleiotropy indicated by a single asterisk. Cases of complete pleiotropy indicated by two asterisks. Dashes are associated with incalculable parameter estimates.

[d]Probability of hypothesis (as indicated in parentheses) given pedigree structure with values p<0.050 bolded. Dashes are associated with incalculable parameter estimates.

[e]Maximum-likelihood estimate of environmental correlation. Dashes are associated with incalculable parameter estimates.

[f]Maximum-likelihood estimate of derived phenotypic correlation. Dashes are associated with incalculable parameter estimates.
